# Relationship between Allelic Heterozygosity in *BoLA-DRB3* and Proviral Loads in Bovine Leukemia Virus-Infected Cattle

**DOI:** 10.3390/ani11030647

**Published:** 2021-03-01

**Authors:** Hala El Daous, Shuya Mitoma, Eslam Elhanafy, Huyen Thi Nguyen, Ngan Thi Mai, Kosuke Notsu, Chiho Kaneko, Junzo Norimine, Satoshi Sekiguchi

**Affiliations:** 1 Graduate School of Medicine and Veterinary Medicine, University of Miyazaki, 5200 Kihara, Kiyotake-cho, Miyazaki 889-1692, Japan; hala.ali@fvtm.bu.edu.eg (H.E.D.); gf11027@student.miyazaki-u.ac.jp (S.M.); notsu.kousuke.p0@cc.miyazaki-u.ac.jp (K.N.); 2 Faculty of Veterinary Medicine, Benha University, Moshtohor, Toukh, Qalyubia 13736, Egypt; dr.eslam.elshehry@gmail.com; 3 National Institute of Veterinary Research, Hanoi 100000, Vietnam; nguyenhuyen150187@gmail.com; 4 Faculty of Veterinary Medicine, Vietnam National University of Agriculture, Hanoi 100000, Vietnam; mtngan@vnua.edu.vn; 5 Center for Animal Disease Control, University of Miyazaki, 1-1 Gakuen-Kibanadai-Nishi, Miyazaki 889-2192, Japan; ckaneko@cc.miyazaki-u.ac.jp (C.K.); nori@cc.miyazaki-u.ac.jp (J.N.); 6 Department of Veterinary Science, Faculty of Agriculture, University of Miyazaki, 1-1 Gakuen-Kibanadai-Nishi, Miyazaki 889-2192, Japan

**Keywords:** *BoLA-DRB3* allele combinations, bovine leukemia virus, heterozygous alleles, proviral load, RFLP-PCR

## Abstract

**Simple Summary:**

Bovine leukemia virus (BLV) caused a severe cattle neoplastic disease called enzootic bovine leukosis (EBL). EBL causes significant economic losses in farming by reducing milk production, reproductive performance, and fertility, and through cattle culling or death. The BLV proviral load (PVL) represents the quantity of BLV genome that has integrated into the host’s genome in BLV-infected cells. It has been reported that PVLs differ according to the genetic background of the host, and some studies of BLV-associated host factors have reported on polymorphisms within the bovine major histocompatibility complex (MHC), namely bovine MHC is bovine leukocyte antigen (*BoLA-DRB3*). However, there is a great diversity in the PVLs associated with carrying various combinations of these alleles, especially for heterozygous alleles. Therefore, this research investigated whether heterogeneity in *BoLA-DRB3* allele combinations would affect PVLs during BLV infections in different ages and breeds of cattle in Japan. This is the first report where the association between heterozygous allelic combinations and BLV PVLs phenotypes (HPLs, LPLs) was analyzed. Our findings augment current understanding about the immunological role played by *BoLA* heterozygosity in BLV-associated PVLs and biocontrol in BLV infections.

**Abstract:**

Enzootic bovine leukosis is a lethal neoplastic disease caused by bovine leukemia virus (BLV), belongs to family Retroviridae. The BLV proviral load (PVL) represents the quantity of BLV genome that has integrated into the host’s genome in BLV-infected cells. Bovine leukocyte antigen (*BoLA*) class II allelic polymorphisms are associated with PVLs in BLV-infected cattle. We sought to identify relationships between *BoLA-DRB3* allelic heterozygosity and BLV PVLs among different cattle breeds. Blood samples from 598 BLV-infected cattle were quantified to determine their PVLs by real-time polymerase chain reaction. The results were confirmed by a BLV-enzyme-linked immunosorbent assay. Restriction fragment length polymorphism-polymerase chain reaction identified 22 *BoLA-DRB3* alleles. Multivariate negative binomial regression modeling was used to test for associations between BLV PVLs and *BoLA-DRB3* alleles. BoLA-DRB3.2*3, *7, *8, *11, *22, *24, and *28 alleles were significantly associated with low PVLs. BoLA-DRB3.2*10 was significantly associated with high PVLs. Some heterozygous allele combinations were associated with low PVLs (*3/*28, *7/*8, *8/*11, *10/*11, and *11/*16); others were associated with high PVLs (*1/*41, *10/*16, *10/*41, *16/*27, and *22/*27). Interestingly, the BoLA-DRB3.2*11 heterozygous allele was always strongly and independently associated with low PVLs. This is the first reported evidence of an association between heterozygous allelic combinations and BLV PVLs.

## 1. Introduction

Enzootic bovine leukosis (EBL), a lethal neoplastic disease caused by bovine leukemia virus (BLV), belongs to the Retroviridae family (genus *Deltaretrovirus*) [1]. BLV infection has three stages: the silent aleukemic stage; the persistent lymphocytosis (PL) stage, which is characterized by the polyclonal expansion of non-neoplastic CD5+ B-lymphocytes; and the EBL stage, as manifested by malignant CD5+ B-cell lymphoma [2]. While approximately 30% of BLV-infected cattle progress to the PL stage, less than 5% of them develop fatal B-cell lymphosarcoma after a long latent period [3]. EBL causes significant economic losses in farming by reducing milk production, reproductive performance, and fertility, and through cattle culling or death [4]. The proviral load (PVL) of BLV represents the quantity of its genome that has integrated into the genomic DNA of BLV-infected cells. BLV infected animals with high levels of PVL is most likely to develop malignant CD5+ B-cell lymphoma. Thus, PVL is an important index for estimating the stage of a BLV infection in a susceptible animal because it is associated with disease progression, making it a useful way of determining BLV transmission [5].

However, it has been reported that PVLs differ according to the genetic background of the host, and some studies of BLV-associated host factors have reported on polymorphisms within the bovine major histocompatibility complex (MHC) [6,7,8,9]. The bovine MHC, bovine leukocyte antigen (*BoLA*), which is located on chromosome 23, includes three DRB class II genes. *BoLA-DRB3* is highly polymorphic with 130 described alleles and is the only active one of the three DRB genes [10]. Polymorphisms in *BoLA-DRB3* are reportedly associated with susceptibility to several infectious diseases (e.g., BLV-induced lymphocytosis, mastitis, and dermatophilosis) [11]. However, there is a great diversity in the PVLs associated with carrying various combinations of these alleles, especially for heterozygous alleles. Therefore, this research investigated whether heterogeneity in *BoLA-DRB3* allele combinations would affect BLV viral loads during BLV infections in different ages and breeds of cattle in Japan.

## 2. Materials and Methods

### 2.1. Blood Samples

Peripheral blood samples (n = 598) were collected in EDTA tubes from different cattle breeds from four commercial beef farms in Kyushu, Japan. These farms raised mainly Japanese Black cattle (523), Holstein cattle (68), Crossbreed (4) and Jersey (3) (Table 1). All animals recruited in this study were apparently healthy without showing any clinical signs. The samples were collected in January to December 2019. DNA was extracted from 300 µL of each blood sample within a week of its collection using the Wizard^®^ Genomic DNA Purification Kit (Promega, Madison, WI, USA) according to the manufacturer’s instructions. Purified DNA samples were each suspended in 50 µL of distilled water. Plasma was separated from 1000 µL of each blood sample by centrifugation (1500× *g*, 5 min, 4 °C) and then stored at −20 °C until analysis.

### 2.2. Real-Time PCR Quantification of BLV-Associated PVLs

The concentration and purity of each DNA sample was determined with a NanoDrop 8000 spectrophotometer (Thermo Fisher Scientific, Wilmington, DE, USA), and each sample was diluted to 20 ng/µL. Proviral loads were quantified on the Applied Biosystems 7300 Real-Time PCR System (Applied Biosystems, CA, USA). The reaction mixture contained 5 µL of 2× Cycleave PCR^®^ Reaction Mix SP (Takara-Bio Inc., Shiga, Japan), 0.2 µL of probe/primer mix for BLV, 0.1 µL of ROX™ Reference Dye II (Takara-Bio Inc., Shiga, Japan), 1 µL of DNA template (20 ng/µL), and PCR-grade water to a final 10 µL volume. To determine PVLs, calibration curves were generated from the measured concentrations of a dilution series of positive control DNA containing the BLV TAX gene (Takara-Bio Inc., Shiga, Japan) and the PVL was expressed as the number of provirus copies/50 ng of genomic DNA [12].

### 2.3. Diagnosing BLV Infections Using BLV-Enzyme-Linked Immunosorbent Assay (ELISA)

All plasma samples were analyzed using a BLV gp51 antibody detection ELISA kit (JNC, Tokyo, Japan) according to the manufacturer’s instructions.

### 2.4. Detecting Resistant and Susceptible BoLA-DRB3 Alleles

Restriction fragment length polymorphism-polymerase chain reaction (RFLP-PCR) was used to type the DRB3 alleles from each animal, as previously described [13]. Briefly, for the first amplification round, primers HL030 (5′-ATCCTCTCTCTGCAGCACATTTC-3′) and HL031 (5′-TTTAAATTCGCGCTCACCTCGCCGCT-3′) were used to amplify DRB3 exon 2. For the second amplification round, primers HL030 and HL032 (5′-TCGCCGCTGCACAGTGAAACTCTC-3′) were used for hemi-nested PCR to increase the yield and specificity of the PCR product, which was then used for RFLP-PCR analysis [8]. The first PCR round was performed in a 20 µL reaction mixture containing 1 µL of template DNA (stock DNA), 2 µL of 10× Ex Taq^®^ buffer (Takara-Bio Inc., Shiga, Japan), 1.6 µL of dNTP Mix (Takara-Bio Inc., Shiga, Japan), 0.2 µL of HL030 and HL031 primers (10 pmol each), 0.2 µL of TaKaRa Ex Taq^®^ HS (Takara-Bio Inc., Shiga, Japan), and PCR-grade water up to 20 µL. PCRs involved an initial 2 min denaturation at 98 °C, followed by 10 cycles of 10 s at 98 °C, 15 s at 60 °C, 30 s at 72 °C, and 7 min at 72 °C. The second PCR round was performed in a 40 µL reaction mixture containing 2 µL of template DNA (amplicon from the first PCR round), 4 µL of 10× Ex Taq^®^ buffer (Takara-Bio Inc., Shiga, Japan), 3.2 µL of dNTP mix (Takara-Bio Inc., Shiga, Japan), 0.2 µL of HL030 and HL032 primers (10 pmol each), 0.2 µL of TaKaRa Ex Taq^®^ HS (Takara-Bio Inc., Shiga, Japan), and PCR-grade water to 40 µL. PCR involved an initial 2 min denaturation at 98 °C, followed by 35 cycles of 10 s at 98 °C, 15 s at 60 °C, 30 s at 72 °C, and 7 min at 72 °C. PCR products were analyzed on 2% agarose gels. The second-round PCR amplicons (10 µL each) were digested for 6 h at 37 °C with RsaI and HeaIII, also digested with 5U of BstYI for another 5 h at 60 °C (total volume, 15 µL). Their restriction patterns were obtained by 6% polyacrylamide gel electrophoresis. The nomenclature used for *BoLA-DRB3* alleles is defined in the PCR-RFLP method reported previously [14], as based on the locus. exon* RFLP type format.

### 2.5. BoLA-DRB3 exon2 Cloning and Sequence Analysis

Ambiguous RFLP-PCR results were subjected to sequence analysis verification. Each amplicon extracted from an agarose gel using the QIAquick Gel Extraction Kit (QIAGEN, Hilden, Germany) was cloned into T-vector pMD20 (Takara-Bio Inc., Shiga, Japan), and sequenced. Sequencing was performed in both directions using M13 forward and reverse primers with the Big Dye^®^ Terminator v3.1/1.1 cycle sequencing kit (Applied Biosystems). The resulting data were analyzed by the Applied Biosystems 3730 DNA Analyzer.

### 2.6. Statistical Analyses

Hierarchical clustering in R (version 3.2.2, https://cran.ism.ac.jp/bin/windows/base/old/3.2.2/ (accessed on 14 August 2015)) by Euclidean distance calculation was used to statistically group the BLV PVLs into the following three clusters: the high PVL group (HPL), the medium PVL group (MPL), and the low PVL group (LPL). *BoLA-DRB3* allele frequencies were calculated. Spearman’s rank correlation rho test was conducted on BLV PVLs versus cattle age. Pairwise Wilcoxon rank sum testing was conducted to compare the BLV PVLs among the cattle breeds. The mean PVL for each allele combination was calculated on a heatmap. Violin plots were used to show the BLV PVL distribution patterns for each *BoLA-DRB3* allele combination. Associations between the different *BoLA-DRB3* alleles and BLV PVLs were estimated using the Poisson regression model. The Poisson distribution assumes that the expected and variance values are equal, but this is not always true, causing dispersion of the data when the variance is higher than average. When overdispersion occurs, one of the ways to estimate its parameter is to use the negative binomial distribution [15], which is why we used this regression model in the (MASS) package (http://www.stats.ox.ac.uk/pub/MASS4/ (accessed on 5 May 2003)). The BLV PVL was the outcome, and *BoLA-DRB3* alleles, cattle age and breed were the explanatory variables. Regression model is conducted into two steps: Univariate regression analysis which used for all variables (BoLA-DRB3 alleles, cattle age, and breed). Any variables significantly associated with BLV PVLs at the *p* < 0.20 level were subsequently selected for multivariate regression analysis. The final model was obtained with *p* levels for the remaining variables of <0.05 [16]. Odds ratios (ORs) were calculated using https://www.medcalc.org/calc/odds_ratio.php (accessed on 1 April 2017) to define the BLV susceptible and resistant alleles in the cattle, and *p*-values of less than 0.05 were considered statistically significant. It was reported that MPL and LPL groups were a low-risk spreaders group and HPL was a high-risk spreaders group. They also assumed that if OR > 1 it will be as resistant allele, OR < 1 it will be as susceptible allele [17].

## 3. Results

### 3.1. Quantifying BLV-Associated PVLs and Grouping Cattle According to These Loads

**Figure 1:**
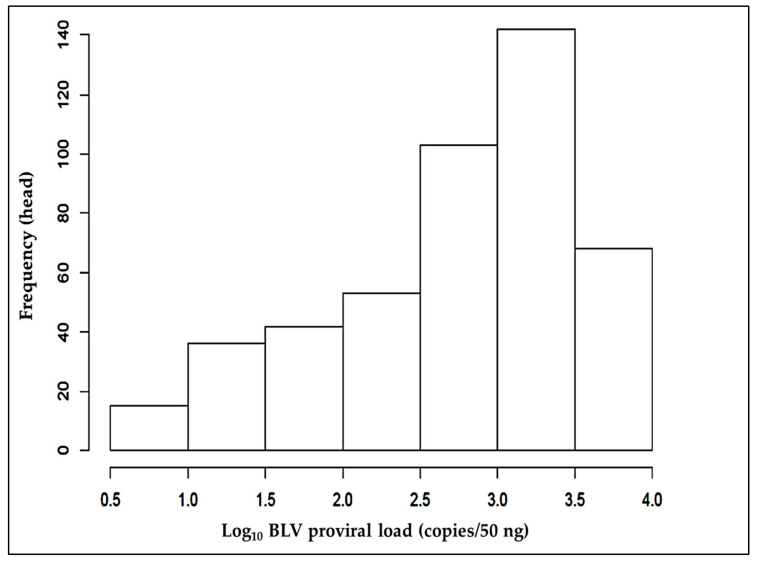
Histogram (log_10_) showing the PVLs associated with BLV infections in the study cattle population. The histogram graphically summarizes and displays the frequencies and left skewed distribution of the log_10_ BLV PVLs (copies/50 ng).

Plasma samples (598) from cattle were confirmed to be BLV-positive without showing of any clinical signs through anti-gp51 antibody detection using BLV-ELISA. The BLV PVLs in peripheral blood mononuclear cells from these cattle were quantified by real-time quantitative PCR, as described earlier. Real-time PCR produced negative results for the 119 samples deemed positive by BLV-ELISA. The PVLs for BLV in terms of copies/50 ng were as follows: minimum 5.0 (log_10_ 0.699), median 767.8 (log_10_ 2.885), and maximum 9107.0 (log_10_ 3.959) (Figure 1). The samples fell into three distinct groups based on the Euclidean distance calculation between each BLV PVL in R by hierarchal clustering. Table 2 shows their statistical grouping (278 HPL, 62 MPL, and 258 LPL).

### 3.2. BLV PVLs and Their Relationships with Cattle Age and Breed

**Figure 2:**
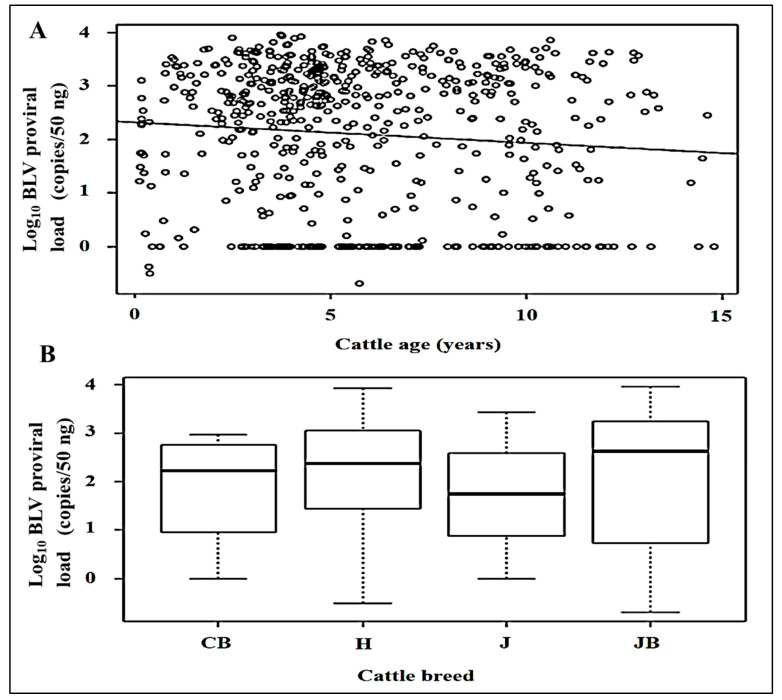
Relationship between PVL, cattle breeds and age. (**A**) Correlation between cattle age (years) and log_10_ BLV PVL (copies/50 ng), adjusted R2 = 0.007. (**B**) Multiple comparison analysis of the different cattle breeds and BLV PVLs. CB: Crossbreed, H: Holstein, J: Jersey, JB: Japanese Black.

The study cattle population comprised two main breeds (Holstein and Japanese Black), and various ages. Therefore, we investigated the relationships between PVL, and the different cattle breeds and ages. There was no significant correlation between BLV PVLs and cattle age (Figure 2A). When we conducted comparison between the BLV PVLs in the different cattle breeds, no significant difference between different cattle breeds and BLV PVLs was found (Figure 2B).

### 3.3. Relationships among BLV and BoLA-DRB3 Alleles, Age, and Breed

**Figure 3:**
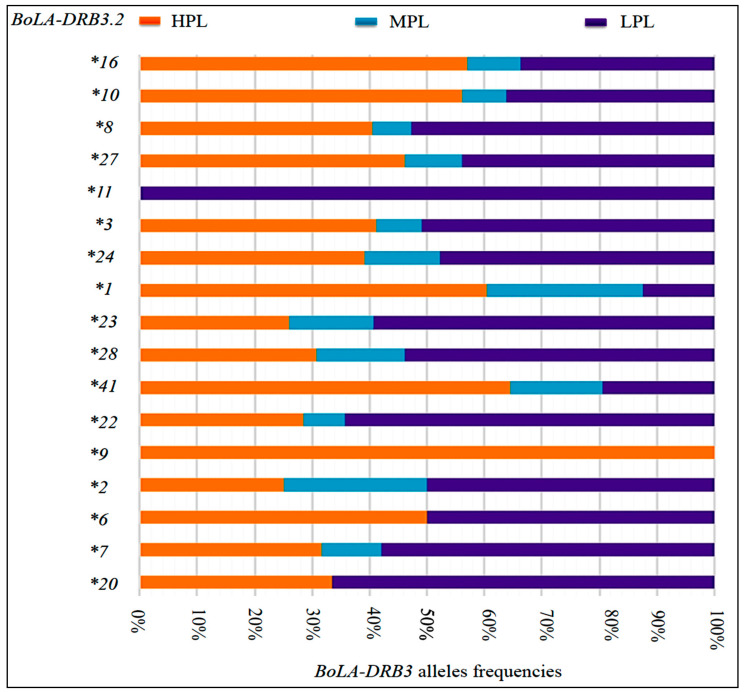
*BoLA-DRB3* allele frequencies in high, medium, and low PVL groups. Graph showing the transition of highly frequent *BoLA-DRB3* alleles from 598 cattle with different BLV PVLs, as genotyped by RFLP-PCR. LPL: low PVL group, MPL: medium PVL group, HPL: high PVL group.

We conducted RFLP-PCR typing of *BoLA-DRB3* alleles to define their relationship with the different BLV PVL groups. Twenty-two different *BoLA-DRB3* alleles were identified in the 598 cattle samples. Table 3 shows the defined allele frequencies and median values for log_10_ BLV PVL versus each *BoLA-DRB3* allele. Based on these results we showed each allele frequencies among the main two cattle breeds in this study (Japanese Black (JB) and Holstein (H)). *BoLA-DRB3*.2*3, *8, *11, *24, *28 were more frequent among JB) than H. While *BoLA-DRB3*.2*7 was only appeared as a heterozygous allele in JB cattle. However, *BoLA-DRB3*.2*22 was more frequent among H than JB (Table S1). All these previously mentioned alleles were categorized as belonging to LPL, whereas *BoLA-DRB3*.2*1, *10, *16, and *41 belonged to HPL (Figure 3). *BoLA-DRB3*.2*1 and *BoLA-DRB3*.2*41 were only appeared in JB cattle. However, *BoLA-DRB3*.2*10 and *BoLA-DRB3*.2*16 were more frequent among JB than H (Table S1). Overall, the inbreeding of the Japanese Black and Holstein herds may have a greater interfere with the prevalence of specific alleles.

Associations between PVL in BLV infections and different *BoLA-DRB3* alleles, age, and breeds were estimated using a univariate negative binomial regression model. The calculated confidence intervals (95%) of the log_10_ PVL median values of all the *BoLA-DRB3* alleles showed the wide range and diversity of the BLV-associated PVLs for each allele from the different combinations, especially the heterozygous ones (Table 3). Multivariate negative binomial regression modeling and OR calculations were conducted for the BLV PVLs and the different *BoLA-DRB3* alleles to define which cattle carried BLV susceptible (OR < 1) or resistant alleles (OR > 1). Table 4 shows that *BoLA-DRB3*.2*11, *3, *7, *8, *22, *28, *20, and *24 alleles were associated with LPLs and MPLs in BLV infections, whereas BoLA-DRB3.2*10 was only associated with HPLs in the infections. Statistically significant allele frequencies were calculated for the different cattle breeds.

### 3.4. Analysis of the Relationship between BoLA-DRB3 Allele Combinations (Heterozygosity) and BLV-Associated PVLs

**Figure 4:**
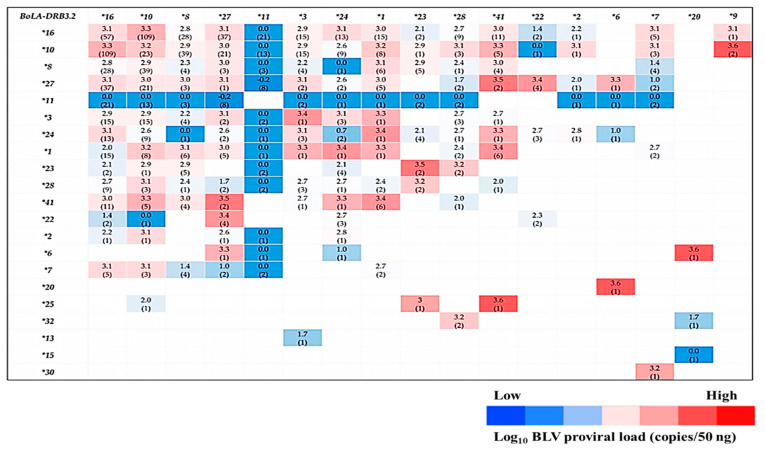
Heat map showing the mean log_10_ values for BLV PVLs (copies/50 ng) and different *BoLA-DRB3* allele combinations. Numbers in each cell indicate the mean values of the different allele combinations, numbers in (): sample numbers for each allele combination. The dark-colored cells show the LPLs in BLV infections and the light-colored cells show the HPLs in these infections. Blank cells represent no samples for this combination.

**Figure 5:**
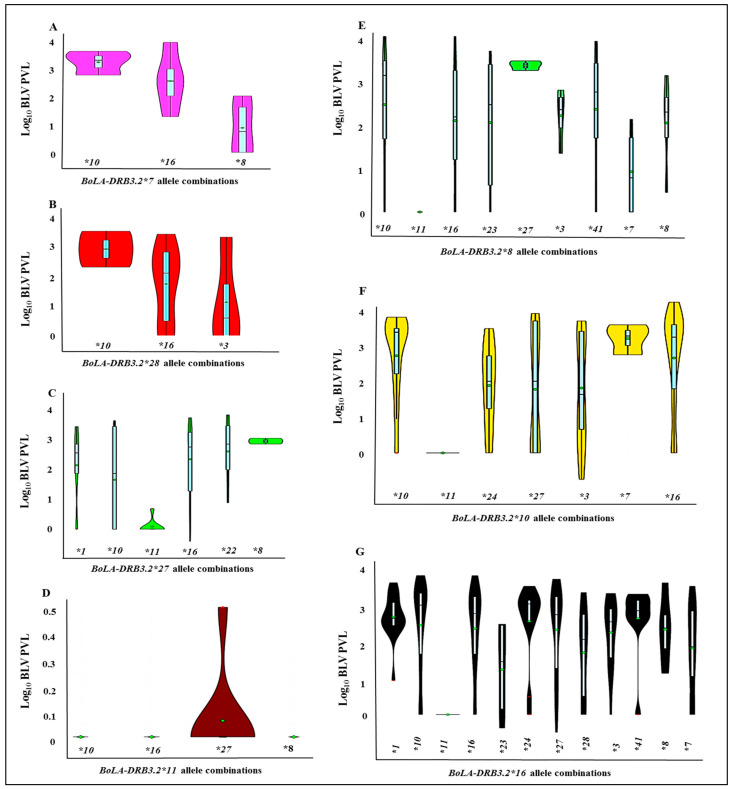
Violin plots (**A**–**G**) show the distribution patterns of Log_10_ BLV PVLs for each *BoLA-DRB3* allele combination. Y axes show log_10_ BLV PVL and X axes show *BoLA-DRB3* allele combinations. (**A**): BoLA-DRB3.2*7 allele combinations, (**B**): BoLA-DRB3.2*28 allele combinations, (**C**): BoLA-DRB3.2*27 allele combinations, (**D**): BoLA-DRB3.2*11 allele combinations, (**E**): BoLA-DRB3.2*8 allele combinations, (**F**): BoLA-DRB3.2*10 allele combinations, and (**G**): BoLA-DRB3.2*16 allele combinations. The green dot represents the median, the thick light blue bar in the center represents the interquartile range, and the thin light blue line represents the rest of the distribution, except for red points that were determined to be “outliers” using a method that is a function of the interquartile range. The colored area on each side of the light blue line is a kernel density estimation to show the distribution shape of the data.

The heat map for the mean values for the log_10_ BLV PVLs was calculated for each *BoLA-DRB3* allele combination (Figure 4). This comprehensive data map led us to statistically evaluate the *BoLA-DRB3* allelic combinations. The resulting violin plots show the distribution patterns of the log_10_ BLV PVLs for the different heterozygous allele combinations. The violin plots in Figure 5A–G indicate that some heterozygous allele combinations are associated with LPLs, while others are associated with HPLs. Specifically, *BoLA-DRB3*.2*11/*16, *10/*11, *3/*28, *8/*11, *7/*8, and *11/*27 allele combinations were associated with LPLs, whereas BoLA-DRB3.2*10/*16, *16/*27, *1/*41, *10/*41, *10/*27, *10/*16, *24/*16, *22/*27, *28/*10, and *28/*16 were associated with HPLs. BoLA-DRB3.2 *16/*1, *16/*41, *1/*10, and *1/*41 were also associated with HPLs (Figure S2).

## 4. Discussion

Our RFLP-PCR genotyping identified 22 previously reported different *BoLA-DRB3* alleles. While our multivariate negative binomial regression modeling and OR calculations showed that BoLA-DRB3.2*11, *8, *28, *7, *3, *24, and *22 alleles were significantly associated with LPLs and MPLs in the BLV infections, the BoLA-DRB3.2*10 allele was significantly associated with HPLs. Nevertheless, the heat map of the log_10_ mean values of the BLV-associated PVLs for each *BoLA-DRB3* allele combination and the violin plots showed the possibility of various allele combinations affecting PVLs in the BLV infections.

*BoLA-DRB3* is a highly polymorphic gene and several of its alleles are favorably or unfavorably associated with BLV-associated PVLs and disease progression. Here, we found that the BoLA-DRB3.2*7 allele was statistically associated with LPLs (*p*-value < 0.01, OR = 1.98), a result supported by a previous study where the BoLA-DRB3*002:01 (DRB3.2*7) allele was reportedly associated with BLV resistance and LPLs (OR > 1) [16]. Surprisingly, we found that BoLA-DRB3.2*7 in combination with BoLA-DRB3.2*10 was associated with HPLs. Our finding that BoLA-DRB3.2*10 is associated with HPLs (OR=0.69) is consistent with the previously reported findings that BoLA-DRB3*016:01 (DRB3.2*10) was associated with HPLs (OR < 1) [17,18]. It is possible that the BoLA-DRB3*016:01 (DRB3.2*10) allele has higher affinity than the BoLA-DRB3*002:01 (DRB3.2*7) allele for antigen presentation to T cell receptors, and as a susceptible allele this causes HPLs. It is also possible that the polymorphisms in the peptide-binding cleft in MHC class II molecules that are responsible for the affinity of the bound peptides influence the magnitude and extent of the elicited adaptive immune response and, therefore, the PVL [19]. High-dose pathogen exposure is also postulated to overcome the immune system, even in the most resistant animals [20]. Monroy et al. reported that the nature of the interaction between two heterozygous alleles combination of the *BoLA-DRB3* gene and BLV resistance or susceptibility is not yet clear [21]. Indeed, although 70% of cattle carrying RR (resistant–resistant) alleles were not infected with BLV, only 33% of SS (susceptible–susceptible) cattle were infected [21]. Moreover, the BoLA-DRB3.2*7–BoLA-DRB3.2*16 combination is associated with a wide and variable PVL range between MPL and HPL. BoLA-DRB3*015:01 (DRB3.2*16) was also determined to be an allele for BLV susceptibility related to HPL (OR < 1) [17]. Furthermore, RR (resistant–resistant) genotypes and SS (susceptible-susceptible) genotypes were found to be associated with LPLs and HPLs, respectively, whereas RS (resistant–susceptible) genotypes covered a wide PVL range [8]. While our results show that the BoLA-DRB3.2*8 allele is statistically associated with LPLs (*p*-value < 0.001, OR = 1.40), elsewhere cattle carrying the BoLA-DRB*012:01 (DRB3.2*8) allele were reported to be at low risk of BLV infection [22]. Thus, we found that BoLA-DRB3.2*7 allele combinations had synergistic effects and were mostly associated with LPLs. Consistent with this finding, a previous report showed that the RR (resistant-resistant) allele combination carried by all the BLV-infected study animals was associated with LPLs [8]. This may also reflect the high degree of plasticity related to the function of different genes and variants of the MHC system together with other immune defense genes.

That we also found that BoLA-DRB3.2*11 was statistically associated with LPLs (*p*-value < 0.0001, OR = 61.42) confirms previous findings that the BoLA-DRB3*009:02 (DRB3.2*11) allele is strongly associated with BLV resistance and LPLs (OR > 1) [9,17,23]. Moreover, our study indicated that the heterozygous BoLA-DRB3.2*11 allele is always strongly and independently associated with very low PVLs, whatever the allele combinations. Cattle carrying the BoLA-DRB3*009:02 (DRB3.2*11) allele may develop strong protective immunity against BLV infection and control the virus by eliciting strong BLV-specific CD4+T lymphocytes, whereas some cattle without this allele may fail to do so, resulting in HPLs and disease progression [9]. The BoLA-DRB3*009:02 (DRB3.2*11) resistance allele encodes a peptide motif (named ER) known to be associated with resistance to persistent lymphocytosis in BLV-infected cattle [13]. Resistance appears to be dependent on the presence of Glu–Arg (polar amino acids) at positions 70–71 within a highly polymorphic segment of its peptide–binding region [13,18]. Therefore, BoLA-DRB3*009:02 (DRB3.2*11) may currently be a candidate marker for BLV resistance in cattle [8].

Interestingly, highly variable PVL ranges have been reported when different allele combinations occur in the presence of heterozygous susceptible *BoLA-DRB3* alleles. BoLA-DRB3.2*10 and *16 are reported to be the susceptible alleles associated with HPLs [17,18,22,23]. Although widely variable PVLs were associated with other described allele combinations (e.g., BoLA-DRB3.2*1, *23, *24, *27, among others) in our violin plots, the homozygous susceptible alleles were mostly associated with HPLs. Heterozygous individuals potentially have a wider variety of effective T lymphocytes than homozygous individuals, which generates a more diverse T-cell repertoire [24]. The susceptible BoLA-DRB3*006:01 (DRB3.2*10) allele, which encodes Glu-Lys (EK) at positions 70 and 71 in the BoLA-DRβ chain, may be associated with HPLs [18]. It has been shown that TNF–α expression levels increased significantly in sheep that were experimentally infected with BLV, and that this was responsible for virus elimination in the early infection stages [25]. When associated with a polymorphism in the MHC class II β chain, polymorphism in the TNF–α promoter gene might influence the host’s response to BLV infection, something that might also occur as a consequence of linkage disequilibrium [8]. In BLV infections, it has been reported that IL-2 production is associated with asymptomatic animals, and that IL-10 production increases in animals with lymphocytosis [26]. These cytokines are mainly produced by activated antigen specific CD4+T lymphocytes, and their activation depends on the presentation of antigenic epitopes by MHC class II molecules. Therefore, it is reasonable to find that certain MHC class II alleles can confer strong protective immunity in BLV-infected animals, whereas others may fail [9].

Another conceivable mechanism controlling PVLs may be related to previously identified haplotypes via the strong linkage between BoLA class II DR and DQ genes [18]. The class II BoLA molecule repertoire is not limited to the intra-haplotype combination of α/β chains; instead, it can be generated by the inter-haplotype combination of α/β chains, as seen among highly polymorphic BoLA DQA (more than two loci) and DQB (more than two loci) genes [27], because these inter-haplotype α/β chain combinations exist with heterozygous BoLA class II alleles in cattle. We hypothesize that the results from the above-mentioned studies indicate that the great diversity seen with BLV-associated PVLs may result from inter-haplotype pairing between the DQA and DQB genes of heterozygous allele combinations. A more detailed study of the linkage between *DR* and *DQ* genes and its effect on BLV PVLs is underway. Further studies in this area are important because it has been reported that many of the genetic and epigenetic factors that are involved in BLV infection exist in addition to *BoLA-DRB3* alleles [8].

We identified various *BoLA-DRB3* allelic types that are associated with MPL or LPL (BoLA-DRB3.2*11, *7, *28, *8, *3, *24, and *22, but not limited to BoLA-DRB3.2*11). The selection of individuals to be parents of the next generation of cattle on the basis of protective alleles is the next logical step in the direction of creating more disease-resistant stock; however, complete information with regards to the different components of *BoLA* such as the haplotype combination of *DQ* and *DR* genes is necessary. It was reported that PVLs vary according to the genetic background of the host, despite having the same clinical status [7]. Thus, in addition to improving production traits, the future application of our findings could also be simultaneously applied to genetic selection as a sort of biocontrol in an integrated strategy. The limitation of this study is that the sample size was not large enough to show significant differences in the BLV PVL values for each heterozygous allele combination.

This is the first report where the association between heterozygous allelic combinations and BLV PVLs (HPLs, LPLs) was analyzed. Our findings show that the heterozygous BoLA-DRB3.2*11 allele is always strongly and independently associated with very low PVLs, whatever the allele combinations. Moreover, allelic divergence and polymorphism in heterozygous allele combinations were found to greatly affect PVLs in BLV infections. Our findings enhance current understanding of the immunological role played by BoLA heterozygosity in BLV-associated PVLs and the biocontrol of BLV infections.

## Figures and Tables

**Table 1:** Summary of the collected samples and their related statistics for age and proviral load (PVL) in the bovine leukemia virus (BLV)-positive cattle.

Cattle Breeds		Number of Collected Samples			
Japanese Black		523			
Farm A		7			
Farm B		10			
Farm C		437			
Farm D		69			
Holstein Farm A		68			
Jersey Farm A		3			
Crossbreed Farm C		4			
Total		598			
Age (years)					
Min.	1st Qu.	Median	Mean	3rd Qu.	Max.
0.1	3.5	5.7	8.0	14.8	4.9

Min.: minimum, Qu.: quarter, Max.: maximum.

**Table 2:** Hierarchical clustering groups for the BLV-associated **log_10_** PVLs based on Euclidean distance calculation.

Clustering Groups of the log_10_ Proviral Load (Copies/50 ng)	Number of BLV-Positive Cattle
LPL grou; <=2.30	258
MPL group; 2.31~2.69	62
HPL group; 2.70~	278
Total	598

PVL: proviral load, LPL: low proviral load, MPL: medium proviral load, HPL: high proviral load.

**Table 3:** *BoLA-DRB3* allele frequencies, univariate negative binomial regression of BLV PVLs and *BoLA-DRB3* alleles, and age and breed of the cattle population.

*BoLA-DRB3* Allele	Allele Frequency	Log_10_ PVL Median (Copies/50 ng)	CI 95%	Regression Coefficients	*p*-Value
DRB3.2* 10	277 (23 Homozygous)	2.8	(0.8–5.4)	Ref.	Ref.
DRB3.2* 16	383 (57 Homozygous)	2.7	(0.8–5.4)	0.091	NS
DRB3.2*8	106 (4 Homozygous)	2.1	(0.5–5.1)	−0.617	**<0.01**
DRB3.2*27	92 (1 Homozygous)	2.6	(0.5–5.6)	−0.134	NS
DRB3.2*11	57	0	(−1–0.3)	−9.343	**<0.0001**
DRB3.2*3	49 (1 Homozygous)	2.4	(0.4–5.4)	−1.0167	**<0.01**
DRB3.2*24	45 (2 Homozygous)	2.6	(0.4–5.4)	−0.764	**<0.05**
DRB3.2*1	49 (1 Homozygous)	2.8	(0.5–5.8)	0.143	NS
DRB3.2*23	21 (2 Homozygous)	2.7	(0.1–6.3)	0.092	NS
DRB3.2*28	28	2.2	(0.2–5.4)	−1.149	**<0.01**
DRB3.2*41	32	2.9	(0.4–6.1)	−0.143	NS
DRB3.2*22	14 (2 Homozygous)	2.3	(−0.1–6.2)	−1.951	**<0.01**
DRB3.2*9	3	3.5	(−0.1–7.1)	0.572	NS
DRB3.2*2	5	2.5	(−0.3–5.7)	−1.215	NS
DRB3.2*6	4	3	(−0.3–6.6)	−0.283	NS
DRB3.2*7	19	1.9	(0.1–5.6)	−1.532	**<0.01**
DRB3.2*20	3	1.7	(−0.6–6.7)	−3.904	**<0.05**
DRB3.2*25	3	3.3	(−1.1–7.6)	1.030	NS
DRB3.2*32	3	3.1	(−1.1–7.3)	0.033	NS
DRB3.2*13	1	1.7	–	−3.278	NS
DRB3.2*15	1	0	–	−23.493	NS
DRB3.2*30	1	3.2	–	0.223	NS
Cattle age	598	−0.015	NS		
Crossbreed	4			Ref.	Ref.
Holstein	68			1.034	NS
Japanese Black	523			1.209	NS
Jersey	3			0.989	NS

PVL: proviral load. Bold means significant *p* value.

**Table 4:** Multivariate negative binomial regression analysis and Odds ratios (ORs) between BLV-associated PVLs and *Bo LA-DRB3* alleles.

*BoLA-DRB3* Allele	Regression Coefficients	*p*-Value	OR	CI95%	Allele Frequencies		
					HPL Group	MPL Group	LPL Group
DRB3.2*10	Ref.	Ref.	0.69	(0.5–0.8)	142	35	100
DRB3.2*11	−9.445 × 10^0^	**<0.0001**	61.42	(8.4–446.7)	0	0	57
DRB3.2*8	−1.319 × 10^0^	**<0.001**	1.40	(0.9–2.2)	43	7	56
DRB3.2*28	−1.094 × 10^0^	**<0.01**	1.89	(0.8–4.6)	8	5	15
DRB3.2*7	−1.681 × 10^0^	**<0.01**	1.98	(0.7–5.0)	6	2	11
DRB3.2*3	−1.055 × 10^0^	**<0.01**	1.33	(0.7–2.4)	20	4	25
DRB3.2*24	−8.777 × 10^−1^	**<0.01**	1.32	(0.7–2.5)	16	7	22
DRB3.2*22	−1.974 × 10^0^	**<0.01**	2.42	(0.7–7.9)	4	1	9

The alleles were determined to be resistant when OR > 1 and susceptible when OR < 1, LPL: low proviral load, MPL: medium proviral load, HPL: high proviral load. Bold means significant *p* value.

## Data Availability

The data presented in this study are available in supplementary martials.

## Supplementary Materials

The following are available online at https://www.mdpi.com/2076-2615/11/3/647/s1, Table S1: *BoLA-DRB3* allele frequencies among the main two cattle breeds in this study Japanese Black (JB) and Holstein (H). Figure S2: Violin plots (H to M) show the distribution patterns of Log10 BLV proviral load (PVL) for each BoLA-DRB3 allele combination. Y axis shows the log10 BLV PVL and the X axis shows the BoLA-DRB3 allele combinations. H: BoLA-DRB3.2*3 allele combinations, I: BoLA-DRB3.2*24 allele combinations, J: BoLA-DRB3*.2*41* allele combinations, K: *BoLA-DRB3.2*22* allele combinations, L: *BoLA-DRB3.2*1* allele combinations, and M: *BoLA-DRB3.2*23* allele combinations. The green dot represents the median, the thick light blue bar in the center represents the interquartile range, and the thin light blue line represents the rest of the distribution, except for the red points determined to be “outliers” using a method that is a function of the interquartile range. The colored area on each side of the light blue line is a kernel density estimation to show the distribution shape of the data.
