# Supplementary material for: Relationship between Allelic Heterozygosity in BoLA-DRB3 and Proviral Loads in Bovine Leukemia Virus-Infected Cattle
Source: Animals (Basel). 2021 Mar 1;11(3):647. doi: 10.3390/ani11030647 (PMC7999362; doi:10.3390/ani11030647)
Supplement: Supplementary file 1 [file animals-11-00647-s001.zip › Table S1.pdf]

Table S1: *BoLA-DRB3* allele frequencies among the main two cattle breeds in this study Japanese Black (JB) and Holstein (H)

***BoLA-DRB3.2* allele**	**Allele frequencies**	
	**Japanese Black (JB)**	**Holstein (H)**
***3**	37	12 (1 homozygous)
***7**	19	-
***8**	99 (4 homozygous)	6
***11**	54	3
***22**	8 (2 homozygous)	6
***24**	26	19 (2 homozygous)
***28**	23	4
***1**	49 (1 homozygous)	-
***41**	32	-
***10**	274 (23 homozygous)	3
***16**	344 (49 homozygous)	39 (8 homozygous)
