# Supplementary material for: Relationship between Allelic Heterozygosity in BoLA-DRB3 and Proviral Loads in Bovine Leukemia Virus-Infected Cattle
Source: Animals (Basel). 2021 Mar 1;11(3):647. doi: 10.3390/ani11030647 (PMC7999362; doi:10.3390/ani11030647)
Supplement: Supplementary file 1 [file animals-11-00647-s001.zip › Figure S1.pdf]

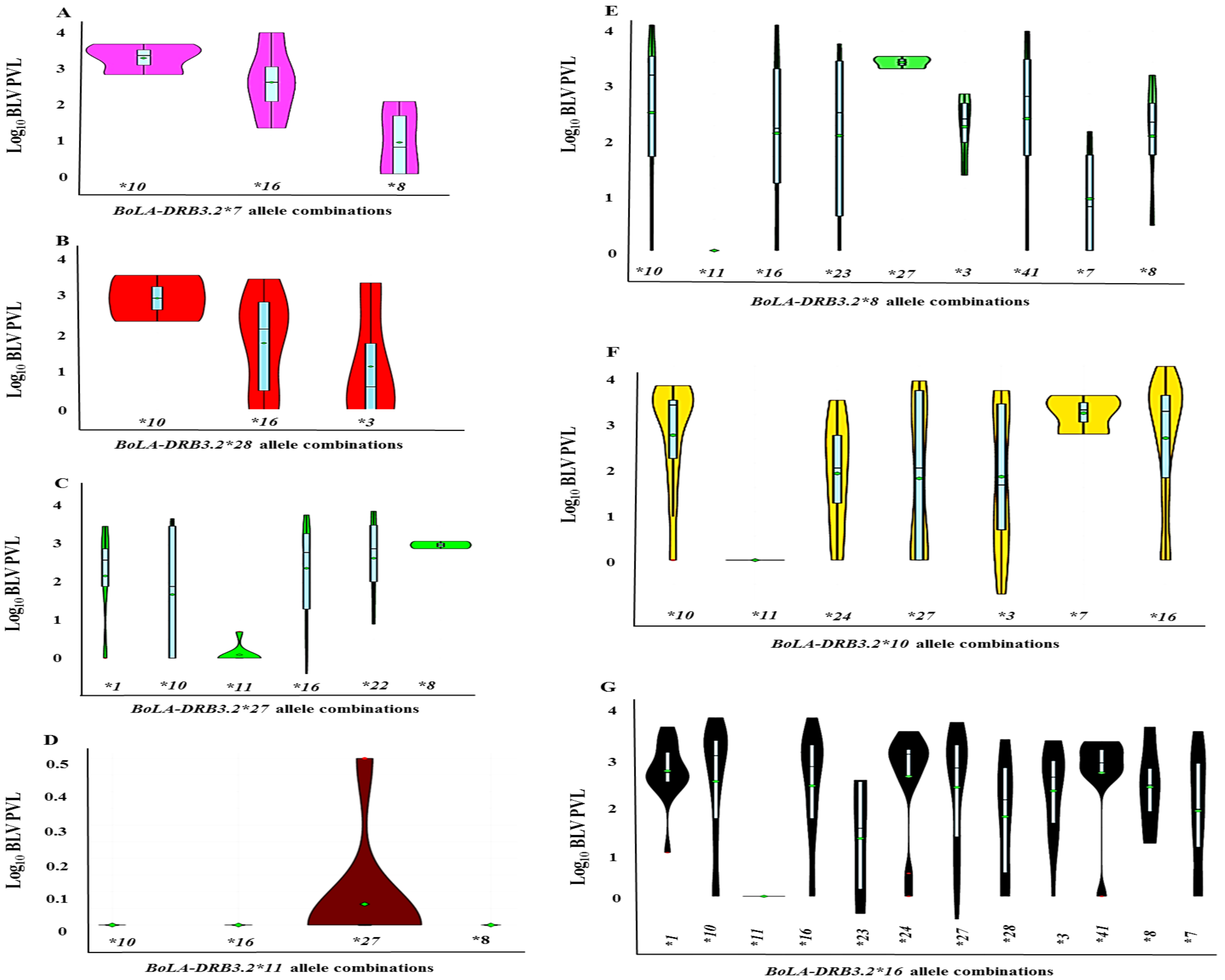
A
Log10 BLV PVL
*10
*16
*8
BoLA-DRB3.2*7 allele combinations
B
*10
*16
*3
BoLA-DRB3.2*28 allele combinations
C
*1
*10
*11
*16
*22
*8
BoLA-DRB3.2*27 allele combinations
D
*10
*16
*27
*8
BoLA-DRB3.2*11 allele combinations
E
*10
*11
*16
*23
*27
*3
*41
*7
*8
BoLA-DRB3.2*8 allele combinations
F
*10
*11
*24
*27
*3
*7
*16
BoLA-DRB3.2*10 allele combinations
G
*1
*10
*11
*16
*23
*24
*27
*28
*3
*41
*8
*7
BoLA-DRB3.2*16 allele combinations

Figure S1: Violin plots (H to M) show the distribution patterns of Log_{10} BLV proviral load (PVL) for each *BoLA-DRB3* allele combination. Y axis shows the log_{10} BLV PVL and the X axis shows the *BoLA-DRB3* allele combinations. H: *BoLA-DRB3.2*3* allele combinations, I: *BoLA-DRB3.2*24* allele combinations, J: *BoLA-DRB3.2*41* allele combinations, K: *BoLA-DRB3.2*22* allele combinations, L: *BoLA-DRB3.2*1* allele combinations, and M: *BoLA-DRB3.2*23* allele combinations. The green dot represents the median, the thick light blue bar in the center represents the interquartile range, and the thin light blue line represents the rest of the distribution, except for the red points determined to be "outliers" using a method that is a function of the interquartile range. The colored area on each side of the light blue line is a kernel density estimation to show the distribution shape of the data.
