# Supplementary figures and images for: Relationship between Allelic Heterozygosity in BoLA-DRB3 and Proviral Loads in Bovine Leukemia Virus-Infected Cattle
Source: Animals (Basel). 2021 Mar 1;11(3):647. doi: 10.3390/ani11030647 (PMC7999362; doi:10.3390/ani11030647)

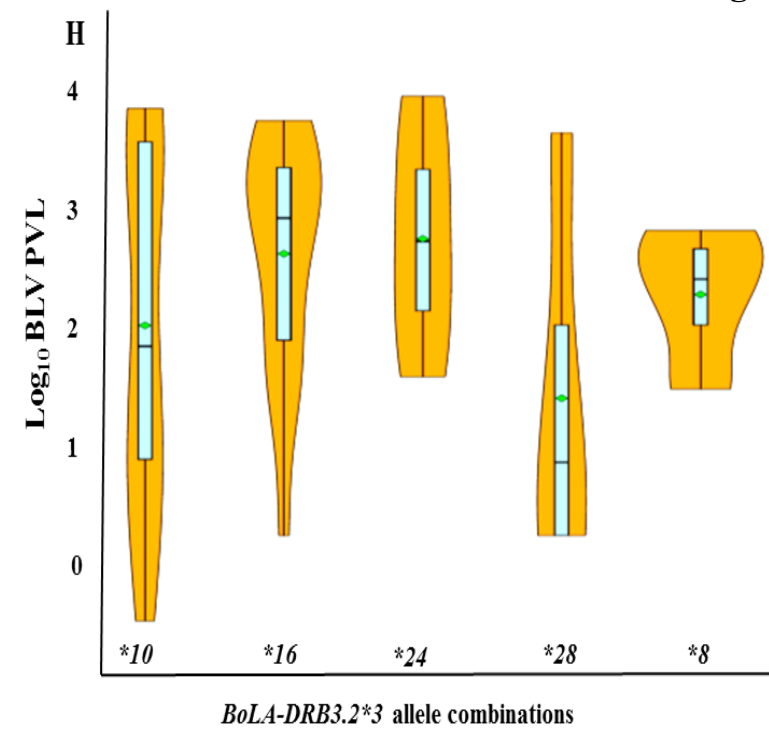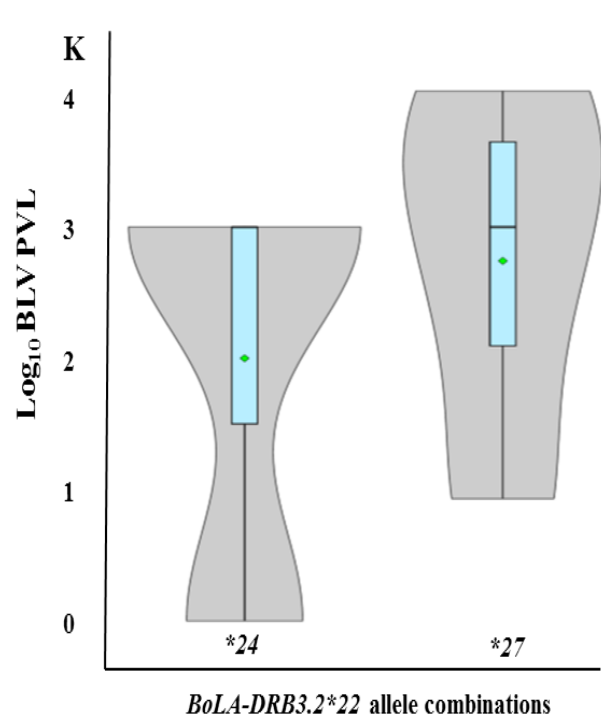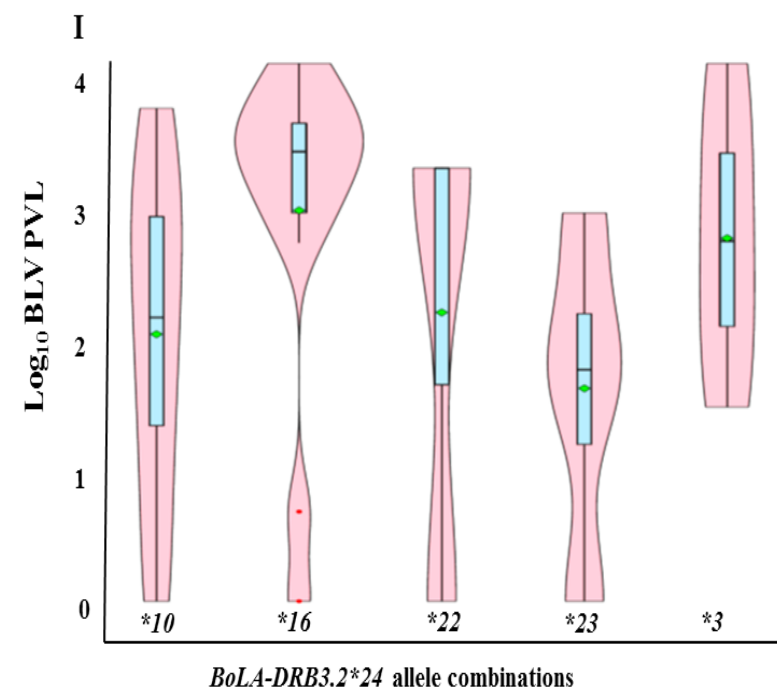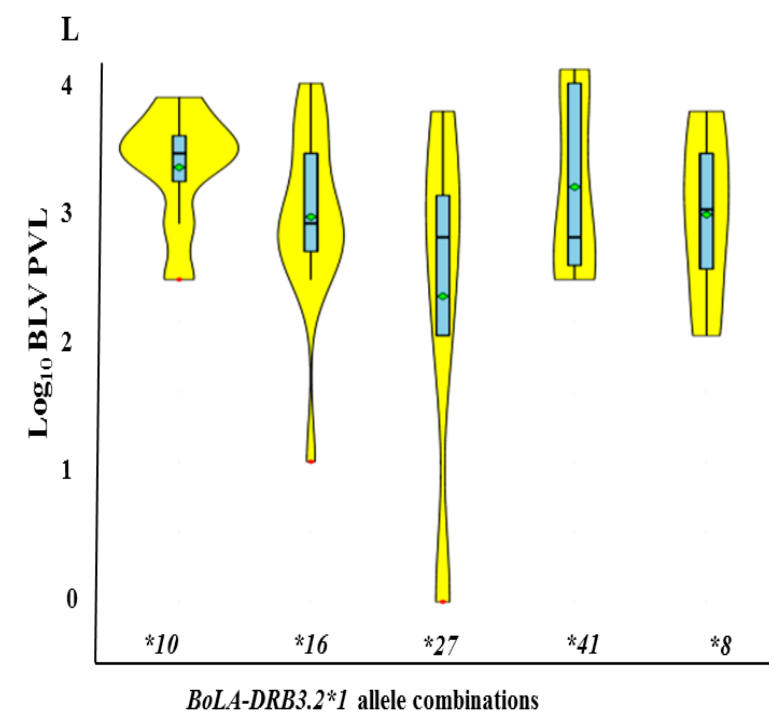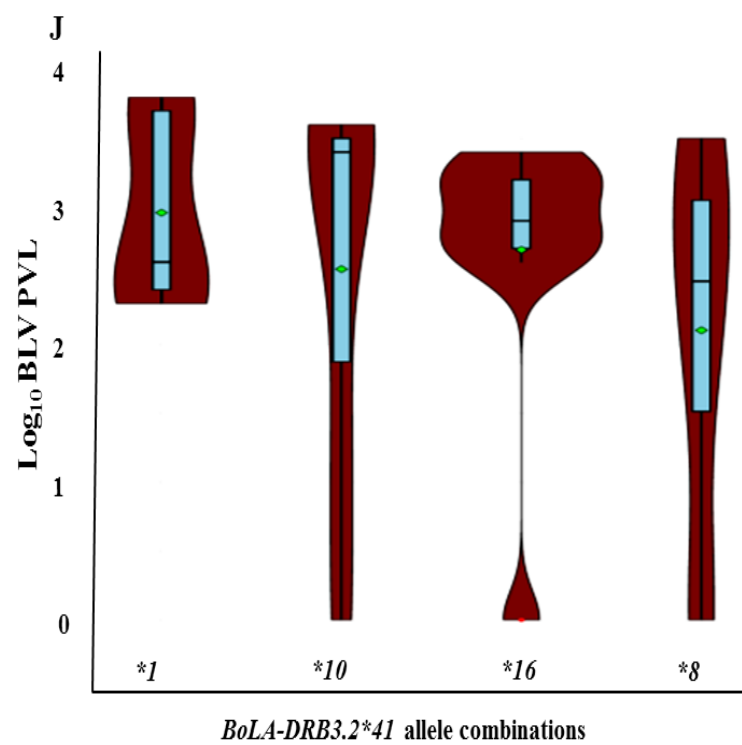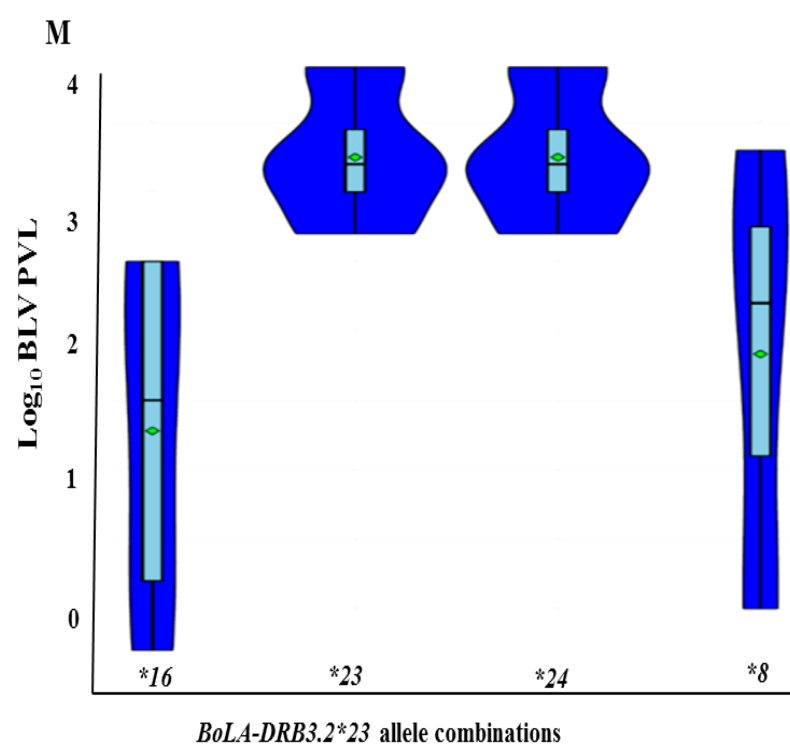

Supplement: Supplementary file 1 [file animals-11-00647-s001.zip › Figure S2 .pdf]
